# Reported daytime sleepiness in relation to orthopnea, restless legs and nocturia in patients evaluated for suspected obstructive sleep apnea

**DOI:** 10.1007/s11325-025-03312-4

**Published:** 2025-03-26

**Authors:** Kristin Marie Hoven, Hans-Jørgen Aarstad, Svein Erik Moe, Sverre K. Steinsvåg

**Affiliations:** 1 Department of Otolaryngology, Head and Neck Surgery, Haukeland University Hospital, 5021 Bergen, Norway; 2 Department of Clinical Medicine, Faculty of Medicine and Dentistry, University of Bergen, Bergen, Norway; 3 Department of Otolaryngology, Head and Neck Surgery, Sørlandet Hospital, 4604 Kristiansand, Norway

**Keywords:** Obstructive sleep apnea, Daytime sleepiness, Nocturia, Orthopnea, Restless legs

## Abstract

**Purpose:**

The aim of the study is to explore the extent to which daytime sleepiness in patients with suspected Obstructive Sleep Apnea (OSA) was correlated with OSA itself and OSA-related comorbidities and symptoms.

**Methods:**

1,305 consecutive patients undergoing OSA workup were included. They underwent standard respiratory polygraphy during sleep and completed a 19-item questionnaire about sleep-related symptoms and signs, as well as the Epworth Sleepiness Scale (ESS). Analyses were based on questionnaire responses and the Apnea–Hypopnea Index (AHI) results and were conducted using stepwise regression analysis.

**Results:**

Using the ESS as the dependent variable, the strongest associations were found with self-reported orthopnea (7%) and restless legs (2%). For daytime sleepiness, self-reported restless legs accounted for 7,6% of the variance, followed by reported orthopnea (3.8%). Regarding daytime irritability, self-reported restless legs accounted for 7.7%, followed by age (4.4%), reported orthopnea (3%), and nocturia (1%) as significant factors. Reported likelihood of falling asleep while driving was best associated with the severity of self-reported restless legs (1,3%), orthopnea (0.6%), and patient age (0.4%). For work performance, restless legs were the strongest predictor (5.9%), followed by age (3.6%) and orthopnea (3%). AHI emerged as a significant explanatory factor regarding ESS score (1.7%) and falling asleep as driver (0.4%) when analyzing the above-mentioned variables.

**Conclusion:**

Daytime sleepiness-associated symptoms were more strongly correlated with reported levels of restless legs, nocturia, and orthopnea than with the AHI score. If restless legs, orthopnea, or nocturia are present, they should be evaluated during the clinical workup for suspected OSA.

**Supplementary Information:**

The online version contains supplementary material available at 10.1007/s11325-025-03312-4.

## Introduction

Obstructive Sleep Apnea (OSA) is a common and serious sleep-related breathing disorder (SRBD) [1]. It is characterized by partial or complete airway collapse during sleep, leading to increased upper airway resistance with a risk of hypopnea or apnea. OSA is defined by an apnea–hypopnea index (AHI) at five or above, with an overall prevalence of 16% in the Norwegian population. When defined by an AHI above 15, the prevalence is 8% [2]. The impact of OSA on health and health-related quality of life can be considerable. Patients with OSA are at an increased risk of coexisting morbidities such as type 2 diabetes, cardiovascular diseases, metabolic syndrome, obesity, cognitive impairment, and chronic fatigue syndrome [3–7]. They are also more frequently involved in driving and workplace accidents [8]. However, if a causative relationship exists with OSA as the contributing factor, it is expected that OSA treatment should reduce the mortality of these coexisting morbidities. This effect has, however, not been demonstrated in asymptomatic patients [1].

Measures against OSA include positional therapy, mandibular advancement devices, nasal corticosteroids, and leukotriene antagonists, with application of Continuous Positive Airways Pressure (CPAP) being the primary treatment for severe OSA [9]. Studies suggest that these measures may also provide benefits such as improved cardiovascular health, increased life expectancy and enhanced quality of life [10]. Additionally, they may help reduce the incidence of traffic and work-related accidents [11].

Poor adherence to CPAP therapy, on the other hand, can limit its effectiveness [12]. Users often report issues such as nasal discomfort, nasal congestion, mask leaking and feelings of claustrophobia. Consequently, the compliance rates varies widely, ranging from 46 to 85% [13]. For those who do not adhere to the treatment symptoms may remain a major problem, affecting personal, social, educational, and professional aspects of life.

Daytime sleepiness has long been considered a key manifestation of OSA, with the Epworth Sleepiness Scale (ESS) being the standard method for evaluation [14]. Published in 1991, the ESS assesses sleepiness in situations where falling asleep is not expected, highlighting negative impacts of inappropriately falling asleep [15]. The association between daytime sleepiness and OSA has been challenged, and currently daytime sleepiness measured by ESS is no longer a prerequisite for being diagnosed with OSA [16]. The ESS questionnaire focuses on excessive daytime sleepiness. Complementing this with questions about daytime alertness could help capture the other end of the sleepiness spectrum, thereby broadening the focus to include daytime tiredness. Such questions could cover aspects like daytime irritability and the impact of tiredness on work performance.

To treat daytime sleepiness, one could furthermore explore physical symptoms often considered secondary to OSA, as some of these symptoms may provide direct clues for treatment options. Nocturia is associated with OSA, but is also linked to urogenital and congestive heart disease [17]. Orthopnea is connected to OSA, but also to heart and lung conditions [18]. Furthermore, restless legs syndrome is related to OSA, but also to various cerebral diseases [19]. Presence of restless legs syndrome may directly contribute to suspected SRBD with daytime sleepiness. Consequently, these conditions could be investigated further to identify additional therapeutic approaches for reducing daytime sleepiness. Treating daytime sleepiness in general better may even reduce mortality, as it is associated with increase mortality risk [20].

The purpose of the present study was to examine daytime sleepiness in patients with suspected OSA from a broader perspective, incorporating questions about daytime functioning. Additionally, the study aimed to explore the potential impact of self-reported nocturia, orthopnea, and restless legs as explanatory factors for various symptoms of daytime sleepiness and tiredness. This approach seeks to expand the information available on suspected OSA patients beyond ESS and AHI scores, with the goal of identifying possible additional treatment strategies.

## Materials and methods

### Study population

The inclusion criteria for this study required that patients had symptoms associated with OSA and were consequently referred to a second line hospital for further investigation. The suspicion of OSA was based on the presence of characteristic symptoms, including nighttime snoring, excessive daytime sleepiness, and reported nighttime episodes of apnea or disrupted breathing during sleep. Exclusion criteria included previous surgery in the nose and pharynx, systemic inflammatory disorders such as Wegener’s granulomatosis and sarcoidosis, drug-induced rhinitis, and current or previous malignancies in the upper airways. Additionally, topical nasal steroids and antihistamines were discontinued one month and one week prior to investigation, respectively.

A total of 1,513 patients referred to the Department of Otolaryngology, Head and Neck Surgery, Sørlandet Hospital, Kristiansand, Norway, were consecutively recruited during the period 2006–2014, after providing informed consent, and underwent a structured diagnostic program. Of these, about 60% were males. The mean age was 46.4 ± 12 years, with an age range from 30 to 81 years. Overall, 1,305 patients with complete data were included in the regression analyses.

The study was approved by the Regional Committee for Medical and Health Research Ethics (REK 2015/1534) with the requirement that patients were informed of the study by mail and given the opportunity to withdraw.

### Study design

This is a monocenter cross-sectional study.

### Respiratory polygraphy

All patients underwent standard respiratory polygraphic recording during sleep using type 3 portable monitors (Embletta or NOX T3, Resmed Norway AS). The following parameters were recorded: plethysmography, nasal flow (using a nasal cannula pressure transducer), SpO_2_, respiratory movements (abdomen and thorax), snoring, ECG, pulse, and position [21]. Scoring was performed in accordance with the 2007 American Academy of Sleep Medicine manual. Apnoea was defined as a reduction of 90% or more in baseline nasal airflow lasting at least 10 s. Hypopnea was defined as a reduction in nasal flow of 30–90% of baseline, also lasting at least 10 s and accompanied by an oxygen desaturation of more than 4%. The diagnosis and severity of OSA were graded based on apnea–hypopnea index (AHI) as follows: no OSA (AHI < 5), mild OSA (AHI 5.0–14.9), moderate OSA (15.0–29.9) or severe OSA (> 30.0) [22]. The baseline data from the respiratory polygraphy are provided in Supplementary Table 1.

### Epworth sleepiness scale (ESS)

The Epworth Sleepiness Scale (ESS) is a simple and rapid method for assessing the degree of daytime sleepiness. It is a patient-reported measure that evaluates sleepiness across eight different everyday situations. The total score ranges from 0 to 24, with higher score indicating a greater degree of sleepiness [23]. All included patients completed the ESS, and a cut-off score of 11 was used to differentiate between normal and excessive levels of daytime sleepiness [24].

### Additional questions

In addition to the ESS, patients also completed a questionnaire addressing comorbidities and symptoms associated with daytime tiredness. The additional questionnaire was in part inspired by the Calgary Sleep Apnea Quality of Life Index [25]. The specific employed questions are listed in Table 1, with responses recorded using a 5-point Likert scale ranging from “Never” to “Always” (Never, Seldom, Sometimes, Often, Always). Patients also provided yes/no responses regarding various medical conditions, including allergies, asthma, rhinitis, diabetes, urogenital disorders, any medication use, as well as information about their alcohol consumption and smoking habits (Table 2).

**Table 1:** Patient-answered questions used as independent and dependent variables

Question number	Question formulation	Question abbreviation
1	Do you wake up with the sensation of not being able to breathe?	Orthopnea
2	Are you sleepy during daytime?	Daytime Sleepiness
3	Do you need to urinate at night?	Nocturia
4	Are your legs restless before you fall asleep?	Restless Legs
5	Have you fallen asleep while driving?	Asleep as Driver
6	Do you feel insufficiently rested when you wake up?	Not Refreshed Morning
7	Are you irritable during the day?	Daytime Irritability
8	Are you tired to the extent that it affects your work performance?	Work Performance
9	Have you been on sick leave due to sleeping difficulties?	Sick Leave Sleepiness
10	How often do you drink alcohol?	Alcohol
11	How often do you snore at night?	Snoring

*English translation of questions originally asked in Norwegian.* The patients answered the questions using a 5-point Likert scale, with responses ranging from “Never” to “Always” (Never, Seldom, Sometimes, Often, Always)

**Table 2:** Patient-reported current comorbidities

Comorbidities	No	Yes
Allergy	1025	428
Asthma	1289	164
Nasal congestion	1038	402
Cardiac diseases	1328	123
Hypertension	1137	313
Kidney disease	1399	51
Use of any medication	671	792
Smoking	943	518

### Statistics

Statistical analyses were performed using a commercially available software package (IBM Corp. Released 2023. IBM SPSS Statistics for Windows, Version 29.0 Armonk, NY: IBM Corp). Statistical significance was defined as *p* < 0.05. All reported* p*-values are from two-sided tests. Pearson correlations coefficients and linear stepwise regression analyses were conducted as indicated.

Independent variables were defined as the patient’s age, self-reported orthopnea, self-reported nocturia, smoking and alcohol history, as well as AHI. Dependent variables were defined to be ESS scores, reported level of not refreshed morning, daytime irritability, asleep as driver, work performance, sick leave sleepiness and information on chronic medical conditions.

## Results

### General patient characteristics

Of the total 1,513 included patients, 428 (29.5%) reported having allergies, 164 (11.3%) had asthma, 402 (27.7%) experienced nasal congestion, 313 (21.5%) had hypertension, 123 (8.5%) had cardiac disease and 51 (3.5%) had kidney disease. Furthermore, 792 (54.1%) reported being on any form of medication, and 518 (35.5%) were currently smoking (Table 2).

### Answering pattern to OSA-related questions

The minimum, maximum, mean, and standard deviation of patient responses to the specific study questions are listed in Table 3.

**Table 3:** Descriptive statistics

Variable	N	Minimum	Maximum	Mean	Standard deviation
Age	1513	10	81	46.4	12.0
Orthopnea	1451	0	4	1.07	1.08
Nocturia	1457	0	4	1.98	1.30
Restless legs	1451	0	4	1.28	1.21
Daytime sleepiness	1457	0	4	2.63	1.03
Not refreshed morning	1454	0	4	2.56	1.11
Daytime irritability	1455	0	4	1.62	0.94
Asleep as driver	1446	0	4	0.32	0.66
Work performance	1437	0	4	1.36	1.01
Sick leave sleepiness	1435	0	4	0.18	0.58
Snoring (self-reported)	1455	0	4	3.29	0.98
Snoring % (measured)	1288	0	93	12.3	15.4
Alcohol	1458	0	4	1.40	0.79
Epworth Sleepiness Scale (ESS)	1464	0	24	10.0	5.20
AHI	1378	0	108	14.6	17.7
ODI	1362	0	128	13.4	17.2
Valid N (list-wise)	1305				

### Correlation between included study variables

Table 4 displays the correlations between the various assigned independent variables. With the exception of reported alcohol consumption, most of the variables showed significant correlations with each other. Table 5 presents the basic correlations between the assigned independent and dependent variables. Notably, only the Epworth Sleepiness Scale scores correlated significantly with the AHI score. Variables such as daytime sleepiness, not feeling refreshed in the morning, and irritability during the day were significantly correlated with most of the other independent variables. Additionally, “Not Refreshed Morning” and “Daytime Irritability” showed some significant correlation with reported alcohol consumption.

**Table 4:** Pearson correlation coefficients between assigned independent variable scores

	Orthopnea	Nocturia	Restless legs	Smoking	Alcohol
Nocturia	.18^***^				
Restless legs	.24^***^	.16^***^			
Smoking	.06^*^	-.09^**^	.08^**^		
Alcohol	-.06^*^	.01	-.02	.13^***^	
AHI	.10^***^	.23^***^	-.04	-.08^**^	-.02

^*^P < 0.05; **P < 0.01; ***P < 0.001

**Table 5:** Pearson correlation coefficients between assigned in dependent (horizontal) and independent (vertical) variable scores

	ESS	Daytime sleepiness	Not refreshed morning	Daytime irritability	Asleep as driver	Work performance	Sick leave Sleepiness	Snoring (Self-reported)
Age	-.02	-.13^***^	-.16^***^	-.21^***^	.08**	-.18^***^	-.12^***^	-.07**
Orthopnea	.27^***^	.27^***^	.25^***^	.23^***^	.13***	.23^***^	.14^***^	.16***
Nocturia	.17^***^	.19^***^	.14^***^	.10^***^	.04	.05	.01	.16***
Restless legs	.20^***^	.26^***^	.28^***^	.27^***^	.11***	.23^***^	.11^***^	.10***
Smoking	-.01	.09^**^	.12^***^	.08^**^	.01	.08^**^	.09^**^	.13***
Alcohol	.04	.04	.07^**^	.05^*^	.03	.03	-.03	.19***
AHI	.15^***^	.04	.03	-.05^*^	.05	-.03	-.04	.13***
Snoring % (measured)	-.01	-.00	.03	.01	-.01	.01	-.05	.15***
BMI	.02	.04	.04	.00	-.00	-.02	-.02	.02

^*^P < 0.05; **P < 0.01; ***P < 0.001

### Linear stepwise regression analyses with ESS, daytime sleepiness, not refreshed morning, daytime irritability or asleep as driver as dependent variables

Table 6 presents the results of linear stepwise regression analyses for the directly sleep-related questions using independent variables such as Epworth Sleepiness Scale, daytime sleepiness, not feeling refreshed in the morning, irritability during the day, and falling asleep while driving.

**Table 6:** Linear stepwise regression analyses

Model	Change statistics			Added predictor
	R^2^ change	F change	Sig. F change	
Epworth Sleepiness Scale (ESS)				
1	.070	95.6	.000	Orthopnea
2	.020	27.6	.000	Restless Legs
3	.017	24.4	.000	AHI
4	.008	11.0	.001	Nocturia
5	.007	10.6	.001	Age
Daytime sleepiness				
1	.076	104.4	.000	Restless Legs
2	.038	54.3	.000	Orthopnea
3	.018	26.8	.000	Nocturia
4	.034	50.6	.000	Age
5	.004	5.7	.017	Smoking
Not refreshed morning				
1	.082	112.8	.000	Restless Legs
2	.035	49.5	.000	Orthopnea
3	.021	31.2	.000	Age
4	.016	24.0	.000	Nocturia
5	.007	9.7	.002	Smoking
Daytime irritability				
1	.077	105.1	.000	Restless Legs
2	.044	62.5	.000	Age
3	.030	44.6	.000	Orthopnea
4	.010	15.4	.000	Nocturia
5	.003	3.9	.049	Asthma
Asleep as driver				
1	.013	17.0	.000	Restless Legs
2	.006	7.9	.005	Orthopnea
3	.004	4.7	.031	Age
4	.004	5.2	.023	AHI

ESS, Daytime Sleepiness, Not Refreshed Morning, Daytime Irritability or Asleep as Driver as dependent variables. Age, gender, smoking, alcohol history, measured physical disease history (Table 2), orthopnea, restless legs and nocturia as independent variables

For ESS as dependent variable, the strongest predictors were reported orthopnea and restless legs, which explained 7% and 2% of the variance, respectively. The AHI score accounted for an additional 1.7% of the variance, with nocturia and patient age contributing an additional 0.8% and 0.7%, respectively.

When analyzing daytime sleepiness and not feeling refreshed in the morning, restless legs emerged as the most significant predictor, accounting for approximately 8% of the variance in both cases. Orthopnea explained around 3.5% of the variance, followed by age, nocturia, and smoking as significant factors.

For daytime irritability, restless legs were again the most significant predictor, explaining 7.7% of the variance, followed by age, orthopnea, and nocturia. Interestingly, asthma contributed uniquely, explaining about 0.5% of the variance.

Regarding the likelihood of falling asleep while driving, the key predictors were restless legs, orthopnea, and age. The AHI score also played a role, accounting for 0.4% of the variance (See Table 6).

### Linear stepwise regression analyses with reported work performance or sick leave sleepiness as dependent variables

Table 7 details the results of linear stepwise regression analyses with work performance and sick leave due to sleepiness as dependent variables.

**Table 7:** Linear stepwise regression analyses with work performance or sick leave sleepiness as dependent variables

Model	Change statistics			
	R^2^ change	F change	Sig. F change	Added predictor
Work performance				
1	.059	77.4	.000	Restless Legs
2	.036	48.6	.000	Age
3	.030	42.6	.000	Orthopnea
4	.008	11.1	.001	Hypertension
5	.004	5.3	.022	Allergy
6	.003	4.4	.037	Nocturia
Sick leave sleepiness				
1	.018	23.2	.000	Smoking
2	.012	15.1	.000	Restless Legs
3	.009	11.3	.001	Age
4	.005	6.5	.011	Orthopnea

Age, gender, smoking and alcohol history, measured physical disease history (Table 2), orthopnea, restless legs and nocturia as independent variables

For reported work performance affected by sleepiness, restless legs were the strongest predictor, explaining 5.9% of the variance. Age and orthopnea followed, explaining 3.6% and 3.0%, respectively. Additional, smaller contributions came from the presence of hypertension (0.8%), allergies (0.4%) and nocturia (0.37%).

Regarding sick leave due to sleepiness, the predictive power of the variables was weaker. The best predictors were smoking (1.8%), followed by restless legs (1.2%), age (0.9%) and orthopnea (0.5%).

### Linear stepwise regression analyses with daytime sleepiness as dependent variable using self-reported snoring (subjective) and measured snoring% (objective) as independent variables

Table 8 displays the results of linear stepwise regression analyses with daytime sleepiness as the dependent variable, examining the impact of both subjective (self-reported) and objective measures (percent of snoring from standard respiratory polygraphic recordings during sleep).

**Table 8:** Linear stepwise regression analyses with daytime sleepiness as dependent variable

Model	Change statistics			
	R^2^ change	F change	Sig. F change	Added predictor
Daytime sleepiness including snoring (self-reported)				
1	.102	161.0	< .001	Snoring
2	.053	89.2	< .001	Restless Legs
3	.030	52.3	< .001	Orthopnea
4	.011	19.8	< .001	Age
5	.019	35.0	< .001	Nocturia
Daytime sleepiness including snoring % (measured)				
1	.076	98.7	< .001	Restless Legs
2	.045	61.3	< .001	Orthopnea
3	.023	32.6	< .001	Nocturia
4	.029	41.8	< .001	Age
5	.004	5.77	.016	Smoking

Age, gender, smoking and alcohol history, measured physical disease history (Table 2), orthopnea, restless legs and nocturia and reported Snoring (self-reported) or measured Snoring % (Measured) as independent variables

When including subjective, self-reported snoring as an independent variable, daytime sleepiness was primarily explained by snoring, accounting for 10% of the variance. Additional predictors included restless legs (5%), orthopnea (3%), age (1.1%) and nocturia (1.9%).

In contrast, when objective snoring percentage was used in the analysis, the strongest predictors were restless legs (7.6%), followed by orthopnea (4.5%), age (2,9%), nocturia (2.3%), and smoking (0.4%), with measured snoring not showing significant association.

### Linear stepwise regression analyses with ESS, daytime sleepiness, not refreshed morning or daytime irritability as dependent variables

Supplementary Table 2 presents the results from linear stepwise regression analyses excluding orthopnea, restless legs and nocturia as independent variables.

For ESS, the AHI score was the strongest predictor, explaining 2.1% of the variance, followed by alcohol use (0.4%) and patient age (0.3%).

Regarding daytime sleepiness, several variables contributed to the variance, including patient age (1.6%), use of any medication (2.0%), nasal congestion (0.9%), hypertension, current smoking, and allergies (0.4% each), and finally the AHI score (0.3%).

For the degree of morning freshness, the results were similar with age (2.0%), use of any medication (1.7%) and smoking (1.3%) being the most significant predictors.

In terms of daytime irritability, age and use of any medication were the top predictors, explaining 1.6% and 2% of the variance, respectively. Nasal congestion, hypertension, smoking, allergies, and AHI score each contributed to less than 1% of the variance. Regarding the likelihood of falling asleep while driving, nasal congestion, AHI score and age each explained about 0.5% (Supplementary Table 2).

### Linear stepwise regression analyses with work performance or sick leave sleepiness as dependent variables

Supplementary Table 2 also shows the results of linear stepwise regression analyses where orthopnea, restless legs, and nocturia were excluded as independent variables.

When analyzing poor work performance due to sleepiness, age emerged as the most significant predictor, explaining 3.6% of the variance. Other contributors include hypertension (1.3%), allergies (0.8%), smoking (0.5%), and the use of any medication (0.3%).

Regarding sick leave due to sleepiness, the most significant predictor was again age, explaining 1.7% of the variance. The use of any medication contributed 0.6%, followed by smoking (0.5%) and allergies 0.3% (Supplementary Table 2).

### Linear stepwise regression analyses with AHI and oxygen desaturation index (ODI) as dependent variables

Table 9 presents the results from linear stepwise regression analyses where AHI or oxygen desaturation index (ODI) were the dependent variables, and BMI, age, gender, use of any medication, nocturia, orthopnea, restless legs, and tobacco use were the independent variables. The analysis revealed that BMI was the strongest predictor in both cases, explaining 5.7% and 7.4% of the variance for AHI and ODI, respectively. Self-reported nocturia was the second strongest predictor, accounting for 4.7 and 3.9%. Patient age was also a predictor in both instances. Additionally, self-reported levels of orthopnea and restless legs were significant predictors, each accounting for between 0.3 and 1% of the variance.

**Table 9:** Linear stepwise regression analyses with Apnea–Hypopnea Index (AHI) or Oxygen Desaturation Index (ODI)HI as dependent variables

Model	Change statistics			
	R^2^ change	F change	Sig. F change	Added predictor
Apnea-Hyponea Index (AHI)				
1	.057	74.4	< .001	BMI
2	.047	63.6	< .001	Nocturia
3	.020	28.8	< .001	Age
4	.005	6.69	.010	Restless Legs
5	.006	9.59	.002	Orthopnea
Oxygen Desaturation Index (ODI)				
1	.074	100.8	< .001	BMI
2	.039	55.2	< .001	Nocturia
3	.011	15.3	< .001	Age
4	.007	9.71	.003	Orthopnea
5	.003	4.24	.040	Restless Legs

BMI, age, gender, use of any medication, restless legs, orthopnea, nocturia and smoking history as independent variables

## Discussion

This report is based on an analysis of self-reported sleep- and tiredness-related symptoms, including the ESS, alongside overnight respiratory polygraphy results from a total of 1,453 patients referred to the hospital with suspected OSA. Self-reported rates of restless legs, orthopnea and nocturia, were statistically and independently associated with reported AHI, ODI and ESS, as well as various symptoms of daytime tiredness. These findings suggest that reported restless legs, orthopnea, and nocturia may be independent risk factors for daytime sleepiness. Furthermore, assessing these factors alongside suspected OSA could enhance the evaluation of daytime sleepiness and tiredness.

Daytime sleepiness implies difficulty in maintaining wakefulness and alertness during the major waking hours of the day. Previously reported independent risk factors from the literature include insomnia, tobacco smoking, anxiety, snoring, somatic symptoms, and obesity [26]. Daytime sleepiness is associated with morbidity and increased mortality [27].

A thorough disease history, physical examination, ESS, and nightly standard respiratory polygraphy recordings are considered key diagnostic elements in the workup of suspected OSA [28]. However, assessing daytime symptoms of tiredness appears to be more complex than merely applying ESS [1]. Clinical research shows that AHI alone has limited ability to predict reduced mortality [29]. This limitation may stem from AHI’s inability to account for important factors such as sleep fragmentation, the duration and distribution of expiratory events, and nocturnal hypoxia [30]. Since daytime tiredness has the potential to reflect these factors [27], it is tempting to consider such symptoms as essential criteria before initiating CPAP treatment, particularly when a relative indication is present.

The present study has emphasized the importance of diagnosing and possibly treating three specific patient-reported conditions, i.e. restless legs, orthopnea, and nocturia to reduce the symptom of daytime sleepiness in conjunction with suspected OSA. Restless legs syndrome (RLS) is a neurological sensory disorder affecting between 5 and 10% of Caucasian populations [31]. It is characterized by a strong urge to move the legs, often accompanied by sensations of itching, tingling, and burning in the lower extremities. These symptoms typically occurs when a person is sitting or lying down, making RLS a potential cause of sleep disturbance and, consequently, daytime sleepiness. A majority of the individuals suffering from RLS also experience periodic limb movements during sleep (PLMS), characterized by involuntary twitching and jerking of the legs, which may further decrease sleep quality and increase daytime sleepiness. Relief measures include iron supplementation, dopaminergic agents, leg movements, massage of the affected area, hot or cold packs and avoidance of alcohol, caffeine, and smoking. Given its impact, RLS and PMLS should be considered when treating patients with daytime sleepiness, OSA and other SRBD. Our study demonstrates that patient-reported severity of restless legs is associated with daytime sleepiness, highlighting it as an important area for treatment in patients with suspected OSA.

Orthopnea is defined as shortness of breath when lying down and is often a symptom of left ventricular failure [32]. In this condition, lying flat increases the load of blood in the pulmonary circulation. While a healthy heart compensates for this redistribution, heart failure leads to blood pooling in the lungs, resulting in shortness of breath, disturbed sleep, and then consequently, daytime sleepiness. Additionally, daytime sleepiness may impair self-management of the heart failure, exacerbating this condition [33]. Orthopnea is associated with OSA. Treatment options may include diuretics, angiotensin-converting enzyme (ACE) inhibitors, and beta-blockers. This study has demonstrated that self-reported orthopnea is associated with self-reported daytime sleepiness, suggesting that this factor should be considered during the evaluation of suspected OSA.

Awakening at least two times per night due to the need to urinate is indicative of nocturnal polyuria, or nocturia, a condition that becomes increasingly common with age. Research shows that over 50% of men and women above 60 years of age suffer from nocturia [34]. This condition is disruptive to sleep and can lead to sleep deprivation. Both our investigation and other studies have found that nocturia is frequently associated with increased daytime sleepiness [35]. Nocturic OSA has even been identified as a distinct phenotype of OSA, characterized by more severe AHI scores, lower oxygen levels, higher body mass index (BMI), and higher scores on ESS [36]. Other contributing factors to nocturia include infections, prostate enlargement, diuretics, diabetes, and neurological disorders [17]. Treatment strategies for nocturia may involve anticholinergic drugs, desmopressin, reducing fluid intake in the hours before bed, and physical therapy to strengthen pelvic muscles. Based on our findings, the diagnosis and treatment of nocturia should be considered with OSA management when these conditions coexist.

We believe that expanding the scope of SRBD investigations could enhance its clinical impact. By exploring not only the negative aspects of tiredness, but also the positive aspects, such as alertness, we can gain a more comprehensive understanding of the issue. Including alertness in assessments also implies that many different conditions may contribute to the overall picture. Our findings suggest that common medical conditions, such as hypertension and allergies, contribute to daytime sleepiness, although their impact appears to be limited. In addition, both alcohol consumption and smoking patterns are contributors. However, psychiatric conditions were not assessed in this study but might play a role [37]. Given the broad range of factors involved, it may be beneficial for the care of SRBD patients to involve collaboration between primary and secondary care providers, ensuring a comprehensive approach to diagnosis and management.

This study combines the analysis of patient-reported data with objective measurements of sleep-related parameters in patients with suspected OSA. However, it is important to acknowledge that patient-reported data may not be as reliable as objective measurements. For instance, in this investigation, the rate of snoring as reported by patients was found to be associated with daytime sleepiness, whereas no such association was observed when using overnight respiratory polygraphy snoring measurements. This discrepancy highlights the need for further investigation and validation of these associations identified in this study to ensure their accuracy and suggested clinical relevance.

The present study is cross-sectional study; therefore, any proposed causal relationships should be interpreted with caution. However, the suggested associations align well with existing knowledge and findings from previous OSA-related research. However, further detailed studies are needed to confirm these associations. Investigations into the effects of therapeutical measures aimed at alleviating nocturia, restless legs and orthopnea on OSA/SRBD will eventually determine their clinical significance.

In conclusion, we have demonstrated an association between self-reported nocturia, orthopnea, restless legs, as well as other medical conditions, alcohol consumption and smoking with daytime sleepiness. These factors should be considered in the workup and treatment of daytime sleepiness, potentially in a collaboration between sleep medicine specialists and primary care physicians.

## Supplementary Information

Below is the link to the electronic supplementary material.

Supplementary file1 (DOCX 19 KB)

Supplementary file2 (DOCX 24 KB)

## Data Availability

Data that support the findings of this study are not openly available due to restrictions set by the Regional Ethical Committee for Medical and Health Research Ethics. However, anonymized data are available from the corresponding author upon reasonable request.
