# Supplementary material for: Reported daytime sleepiness in relation to orthopnea, restless legs and nocturia in patients evaluated for suspected obstructive sleep apnea
Source: Sleep Breath. 2025 Mar 26;29(2):140. doi: 10.1007/s11325-025-03312-4 (PMC11946997; doi:10.1007/s11325-025-03312-4)
Supplement: Supplementary file 1 — Supplementary file1 (DOCX 19 KB) [file 11325_2025_3312_MOESM1_ESM.docx]

| **Variable** | **N** | **Mean (SD)** | **Median (IQR)** |
| --- | --- | --- | --- |
| Participants age (years) | 1513 | 46.4 ± 12 | 46 (38, 55) |
| Body Mass Index (kg/m^2^) | 1469 | 29.2 ± 6.5 | 28.1 (25.7-31.4) |
| Total recording time (minutes) | 1376 | 427.3 ± 69.9 | 426.3 (387.7, 468,3) |
| Apnea-Hypopnea Index (events/hours) | 1378 | 14.6 ±17.7 | 7.6 (2.6, 19,3) |
| Average O2-saturation (%) | 1357 | 94±2.7 | 94.4 (93.1, 95.4) |
| Oxygen Desaturation Index (events/hours) | 1362 | 13.4±17.2 | 6.6 (2.3, 16.8) |
