# Supplementary material for: Reported daytime sleepiness in relation to orthopnea, restless legs and nocturia in patients evaluated for suspected obstructive sleep apnea
Source: Sleep Breath. 2025 Mar 26;29(2):140. doi: 10.1007/s11325-025-03312-4 (PMC11946997; doi:10.1007/s11325-025-03312-4)
Supplement: Supplementary file 2 — Supplementary file2 (DOCX 24 KB) [file 11325_2025_3312_MOESM2_ESM.docx]

| Model | Change Statistics | | | Added Predictor |
| --- | --- | --- | --- | --- |
|  | R^2^ Change | F Change | Sig. F Change |  |
| **Epworth Sleepiness Scale (ESS)** | | | | |
| 1 | .021 | 27.4 | .000 | AHI |
| 2 | .004 | 5.2 | .022 | Alcohol |
| 3 | .003 | 4.5 | .035 | Age |
| **Daytime Sleepiness** | | | | |
| 1 | .016 | 20.2 | .000 | Age |
| 2 | .020 | 26.2 | .000 | Medication |
| 3 | .009 | 11.9 | .001 | Nasal Congestion |
| 4 | .005 | 7.2 | .007 | Hypertonia |
| 5 | .005 | 7.2 | .007 | Smoking |
| 6 | .004 | 5.1 | .024 | Allergy |
| 7 | .003 | 3.9 | .049 | AHI |
| **Not Refreshed Morning** | | | | |
| 1 | .020 | 26.0 | .000 | Age |
| 2 | .017 | 21.9 | .000 | Medication |
| 3 | .013 | 16.7 | .000 | Smoking |
| 4 | .008 | 10.6 | .001 | Nasal Congestion |
| 5 | .004 | 5.8 | .016 | Alcohol |
| 6 | .004 | 5.4 | .021 | Kidney Disease |
| 7 | .004 | 4.8 | .029 | AHI |
| **Daytime Irritability** | | | | |
| 1 | .016 | 20.2 | .000 | Age |
| 2 | .020 | 26.2 | .000 | Medication |
| 3 | .009 | 11.9 | .001 | Nasal cong. |
| 4 | .005 | 7.2 | .007 | Hypertension |
| 5 | .005 | 7.2 | .007 | Smoking |
| 6 | .004 | 5.1 | .024 | Allergy |
| 7 | .003 | 3.9 | .049 | AHI |
| **Asleep as Driver** | | | | |
| 1 | .004 | 4.8 | .028 | Nasal Congestion |
| 2 | .004 | 4.5 | .034 | AHI |
| 3 | .005 | 6.0 | .014 | Age |
| **Work Performance** | | | | |
| 1 | .036 | 46.4 | .000 | Age |
| 2 | .013 | 16.6 | .000 | Hypertension |
| 3 | .008 | 9.9 | .002 | Allergy |
| 4 | .005 | 7.1 | .008 | Smoking |
| 5 | .003 | 4.5 | .035 | Medication |
| **Sick Leave Sleepiness** | | | | |
| 1 | .017 | 21.3 | .000 | Age |
| 2 | .006 | 7.9 | .005 | Medication |
| 3 | .005 | 6.5 | .011 | Smoking |
| 4 | .003 | 4.4 | .036 | Allergy |
